# Sex differences in arterial identity correlate with neointimal hyperplasia after balloon injury

**DOI:** 10.1007/s11033-022-07644-2

**Published:** 2022-06-17

**Authors:** Mingjie Gao, Xixiang Gao, Ryosuke Taniguchi, Anand Brahmandam, Yutaka Matsubara, Jia Liu, Hao Liu, Weichang Zhang, Alan Dardik

**Affiliations:** 1 Department of Vascular Ultrasonography, Xuanwu Hospital, Capital Medical University, Beijing, China; 2 Vascular Biology and Therapeutics Program, Yale School of Medicine, New Haven, CT USA; 3 Division of Vascular and Endovascular Surgery, Department of Surgery, Yale School of Medicine, New Haven, CT USA; 4 Department of Vascular Surgery, Xuanwu Hospital, Capital Medical University, Beijing, China; 5 Division of Vascular Surgery, The University of Tokyo, Tokyo, Japan; 6 Department of Surgery and Sciences, Kyushu University, Fukuoka, Japan; 7 Division of Vascular and Interventional Radiology, Department of General Surgery, Nanfang Hospital, Southern Medical University, Guangzhou, China; 8 Department of Surgery, VA Connecticut Healthcare Systems, West Haven, CT USA; 9 Yale School of Medicine, 10 Amistad Street, Room 437, PO Box 208089, New Haven, CT 06520-8089 USA

**Keywords:** Neointimal proliferation, EphrinB2, Vessel identity, Sex differences, Balloon injury

## Abstract

**Background:**

Endovascular treatment of atherosclerotic arterial disease exhibits sex differences in clinical outcomes including restenosis. However, sex-specific differences in arterial identity during arterial remodeling have not been described. We hypothesized that sex differences in expression of the arterial determinant erythropoietin-producing hepatocellular receptor interacting protein (Ephrin)-B2 occur during neointimal proliferation and arterial remodeling.

**Methods and results:**

Carotid balloon injury was performed in female and male Sprague–Dawley rats without or 14 days after gonadectomy; the left common carotid artery was injured and the right carotid artery in the same animal was used as an uninjured control. Arterial hemodynamics were evaluated in vivo using ultrasonography pre-procedure and post-procedure at 7 and 14 days and wall composition examined using histology, immunofluorescence and Western blot at 14 days after balloon injury. There were no significant baseline sex differences. 14 days after balloon injury, there was decreased neointimal thickness in female rats with decreased smooth muscle cell proliferation and decreased type I and III collagen deposition, as well as decreased TNFα- or iNOS-positive CD68+ cells and increased CD206− or TGM2-positive CD68+ cells. Female rats also showed less immunoreactivity of VEGF-A, NRP1, phosphorylated EphrinB2, and increased Notch1, as well as decreased phosphorylated Akt1, p38 and ERK1/2. These differences were not present in rats pretreated with gonadectomy.

**Conclusions:**

Decreased neointimal thickness in female rats after carotid balloon injury is associated with altered arterial identity that is dependent on intact sex hormones. Alteration of arterial identity may be a mechanism of sex differences in neointimal proliferation after arterial injury.

**Supplementary Information:**

The online version contains supplementary material available at 10.1007/s11033-022-07644-2.

## Introduction

Atherosclerotic peripheral artery disease (PAD) is a major cause of global mortality and morbidity affecting over 200 million people [1]. Endovascular therapy such as balloon angioplasty or stenting is a well-established, minimally invasive treatment for patients with PAD. However, restenosis after endovascular therapy is a major concern, especially in men. Published studies are not consistent in predicting outcomes by sex after endovascular treatment in patients with PAD, with some studies reporting worse outcomes in men [2–4], while others show no sex-related differences [5]. Despite sexual dimorphism in the incidence and complications of restenosis after endovascular therapy, there are relatively limited preclinical data that address mechanisms underlying sex as a biological variable in restenosis [6], and thus mechanisms of sex differences in restenosis after endovascular therapy remain unclear.

The molecular identity of arteries and veins is determined as the circulatory system develops during embryogenesis. We have previously shown that vessel identity can be plastic in adults, with changes in identity occurring with changes in hemodynamic environments [7]. EphrinB2 is a determinant of arterial identity and present on both arterial endothelial and smooth muscle cells [8]; other determinants of arterial identity include the vascular endothelial growth factor-A (VEGF-A) and one of its receptors, VEGF-R2, as well as the Notch family [7]. EphrinB2 is a preferred ligand for erythropoietin-producing hepatocellular B4 (EphB4) and functions as an important regulator of blood vessel formation and remodeling [8, 9]. We have previously shown that activation of EphrinB2 signaling promotes venous remodeling including increased venous and arterial diameter and wall thickness in arteriovenous fistulae [10]. Furthermore, exposure to exogenous 17β-estradiol (E2) during vascular niche development significantly disrupts EphrinB2 arterial identity by decreasing the expression of VEGF-A and its downstream Notch pathway [11]. Since arterial identity can regulate vascular remodeling, and estrogen can regulate arterial identity, we questioned whether there are sex differences in expression of arterial identity during restenosis after arterial injury.

The rat carotid balloon injury model has been used extensively to characterize neointimal proliferation and identify various mechanisms of regulation [12–14]. We hypothesized that sex differences in arterial identity occur during neointimal proliferation and arterial remodeling, suggesting a novel mechanism by which sex differences may regulate the arterial response to injury.

## Materials and methods

### Rat balloon injury model

All animal experiments were performed in accordance with federal guidelines and Yale University Institutional Animal Care and Use Committee approval. Ten-week-old female and male Sprague–Dawley (SD) rats were obtained from Charles River Breeding Laboratories (Wilmington, MA). Females weighed 250–270 g and males weighed 290–310 g. All rats were fed a standard diet (Ralston Purina Diet) ad libitum. Body weights were measured before, at 1 week and 2 weeks after balloon injury of the carotid artery.

Female and male SD rats were randomly divided into two subgroups. The first group comprised rats with intact gonads (n = 12 in each sex) and the second group comprised rats treated with gonadectomy, either bilateral oophorectomy in female rats or castration in male rats (n = 6 in each sex) as previously described [15]. Fourteen days after gonadectomy, female and male rats were treated with carotid artery balloon injury (Fig. S1A). Balloon injury was performed at 12-weeks of age.

Carotid balloon injury was performed as previously described. Briefly, the left carotid artery was injured with the right carotid artery serving as an autologous uninjured control; left carotid artery injury was performed with an inflated balloon that denudes the endothelium and distends the vessel wall [12–14]. All rats were anesthetized using 4% isoflurane in 1.0 L/min oxygen for induction, followed by maintenance with 2% to 3% isoflurane. The left external carotid artery was isolated by a middle cervical incision, suspended on ties, and stripped of adventitia. Balloon injury was performed by introducing a 2F Fogarty balloon catheter (Edwards Lifesciences, Irvine, CA) via a distal branch of the external carotid artery and advanced into the aorta arch. The balloon was inflated with water at physiological pressure (1.5 atm) and passed through the entire common carotid artery three times; after the third pass the catheter was removed and the branch vessel ligated. Of note, the common carotid artery and region of the bifurcation were not surgically manipulated or exposed. Immediate visible pulsatile arterial blood flow in the common carotid artery was used as a marker of patency. The incision was closed, and the animal allowed to recover; postoperative pain control was achieved with intraperitoneal delivery of buprenorphine (0.1 mg/kg). Both the injured and uninjured carotid arteries was harvested at baseline or on postoperative day 14. Only patent carotid arteries were included in this study; a single male rat with carotid artery thrombosis due to technical failure was excluded.

### Ultrasonography

The Vevo 770 High Resolution Imaging System (VisualSonics, Toronto, Ontario, Canada) with a RMV704 (40 MHz) probe was used in the carotid scan mode and a RMV707B (30 MHz) probe was used in the cardiac scan mode. Ultrasound measurements were obtained at the middle segment of the common carotid artery on preoperative days 0, 7 and 14. Quantitative measurements were made at the end-diastolic frames; velocity measurements were made with the angle between the ultrasound beam and the blood flow set at ≤ 60°. Doppler ultrasound was used to measure the common carotid artery outer and inner diameters in cross-sectional views and the neointimal thickness and velocities in longitudinal views, at a distance of 2–4 mm from the carotid bifurcation. The outer diameter was measured from the anterior to the posterior edge of the outer border of the sonolucent zone representing the media-adventitia leading edge, which corresponds to the external elastic lamina in histopathologic specimens; the inner diameter was measured from the anterior to the posterior of the interface between the vessel lumen and the edge of the initial echogenic layer, which corresponds to the intimal-luminal leading edge [16]. The neointimal thickness was measured at the thickest part from the inner edge to the outer edge in the specified measurement area (Fig. S1B).

Analysis of the recorded images was performed offline after the procedure. The resistive index (RI) was calculated using the peak systolic velocity (PSV) and the end diastolic velocity (EDV) of the carotid artery. Shear stress across the carotid artery was calculated by incorporating the carotid diameter and velocity, by the Hagen-Poiseuille formula (Fig. S1C).

### Histology

The animals were euthanized and perfused with normal saline followed by 10% formalin via the abdominal aorta under physiological pressure and the common carotid artery was extracted en bloc. The tissue block was then embedded in paraffin, cut in 5-μm cross sections, with 3 to 5 consecutive sections mounted onto each glass slide. Hematoxylin and eosin (HE), Masson trichrome, and Elastin Van Gieson (EVG) staining were performed. Digital images of these sections were captured using a microscope (BX40; Q-Color 5, Olympus America, Center Valley, PA) and analyzed with ImageJ software (National Institutes of Health, Bethesda, MD). The mean thickness of the intima and media were obtained by the average of measurements from eight equidistant points of the carotid artery [17, 18].

### Immunofluorescence

Unstained cross-sections were deparaffinized with xylene and rehydrated in a graded series of alcohols. Antigen retrieval was performed with citric acid buffer (pH 6.0) for 10 min at 100 °C, followed by blocking with 5% bovine serum albumin for 1 h at room temperature. Subsequently, sections were incubated with primary antibodies (Table S1) overnight at 4 °C. The following day, sections were incubated in secondary antibodies (Table S1) for 1 h at room temperature and counterstained with DAPI (Invitrogen, P36934). For negative controls for the antibodies, IgG isotype controls and endogenous tissue background controls were used. Immunofluorescence images were captured using a fluorescent microscope (Carl Zeiss MicroImaging, Inc.) and quantified using ImageJ software (National Institutes of Health, Bethesda, MD).

### Western blot

The common carotid arteries were removed with care taken to avoid surrounding connective tissues. Proteins were extracted with RIPA buffer containing 0.1% SDS, EDTA-free Protease Inhibitor (Sigma-Aldrich; cOmplete™) and phosphatase inhibitor (Sigma-Aldrich; PhosSTOP™). Protein concentrations were assessed using a colorimetric assay (Bio Rad), and equal amounts of protein were run on a sodium dodecyl sulfate polyacrylamide gel electrophoresis gel; proteins were then transferred to a polyvinylidene difluoride membrane (0.45 μm pore size; Immobilon, Millipore), and blocked in PBST containing 5% BSA for 1 h at room temperature, followed by incubation with primary antibodies (Table S1) overnight at 4 °C on a shaker. After washing the membranes with PBST, incubation with an HRP-linked secondary antibody (Table S1) was performed for 1 h at room temperature, and signals were detected using an ECL substrate reagent (PerkinElmer; NEL105001EA or Thermo Scientific™; 34095) and film processor SRX-101A (Konica Minolta, Ramsey, NJ). Where needed, membranes were stripped with Restore Western Blot Stripping Buffer (Pierce Biotechnology, Rockford, IL) and repeated as described above.

### Statistical analysis

All images were quantified using ImageJ software (National Institutes of Health, Bethesda, MD). All statistical analysis was performed using Prism 8 (GraphPad Software, Inc., La Jolla, CA) and recorded as mean ± standard error of the mean (SEM). Normality was confirmed using the Shapiro–Wilk test. Statistical significance was determined using Student’s t test or ANOVA with Sidak’s post hoc correction. The Mann–Whitney U test or the Kruskal–Wallis test with Dunn post hoc correction was used for non-parametric tests. Patency and survival were analyzed using Kaplan–Meier analysis. *p* < 0.05 were considered significant.

## Results

### Similar baseline carotid artery structure and hemodynamic characteristics

To determine whether there are sex differences in arterial identity during remodeling, female and male SD rats were treated with carotid balloon injury, either without or with pretreatment with gonadectomy. The baseline characteristics of female and male rats were similar except for reduced weights of female rats in both intact and gonadectomy groups (Table S2). The body weight gain of castrated male rats was significantly reduced compared to intact male rats (22 g/week vs. 43 g/week), whereas castrated female rats had increased body weight gain compared to intact female rats (17 g/week vs. 7 g/week; Fig. S2A). Female rats had similar rates of carotid artery patency and postoperative survival after balloon injury compared to male rats in both intact and gonadectomy groups (Fig. S2A).

Ultrasound evaluation of the carotid artery morphology before balloon injury showed a single cell-layer intima, an intact internal elastic lamina and an external elastic lamina that was in contact with the adventitia in all animals of both sexes and groups; there were no differences in the baseline diameter, luminal area or wall thickness among the groups (Figs. S1C, S2B and Table S3), consistent with lack of detectable sex differences in the baseline anatomy of the carotid artery, and without any effect of gonadectomy. Similarly, there were no differences in carotid artery baseline hemodynamics including similar waveforms (Table S3) among all the groups*.*

### Reduced neointimal proliferation after balloon injury in intact female rats

**Fig. 1:**
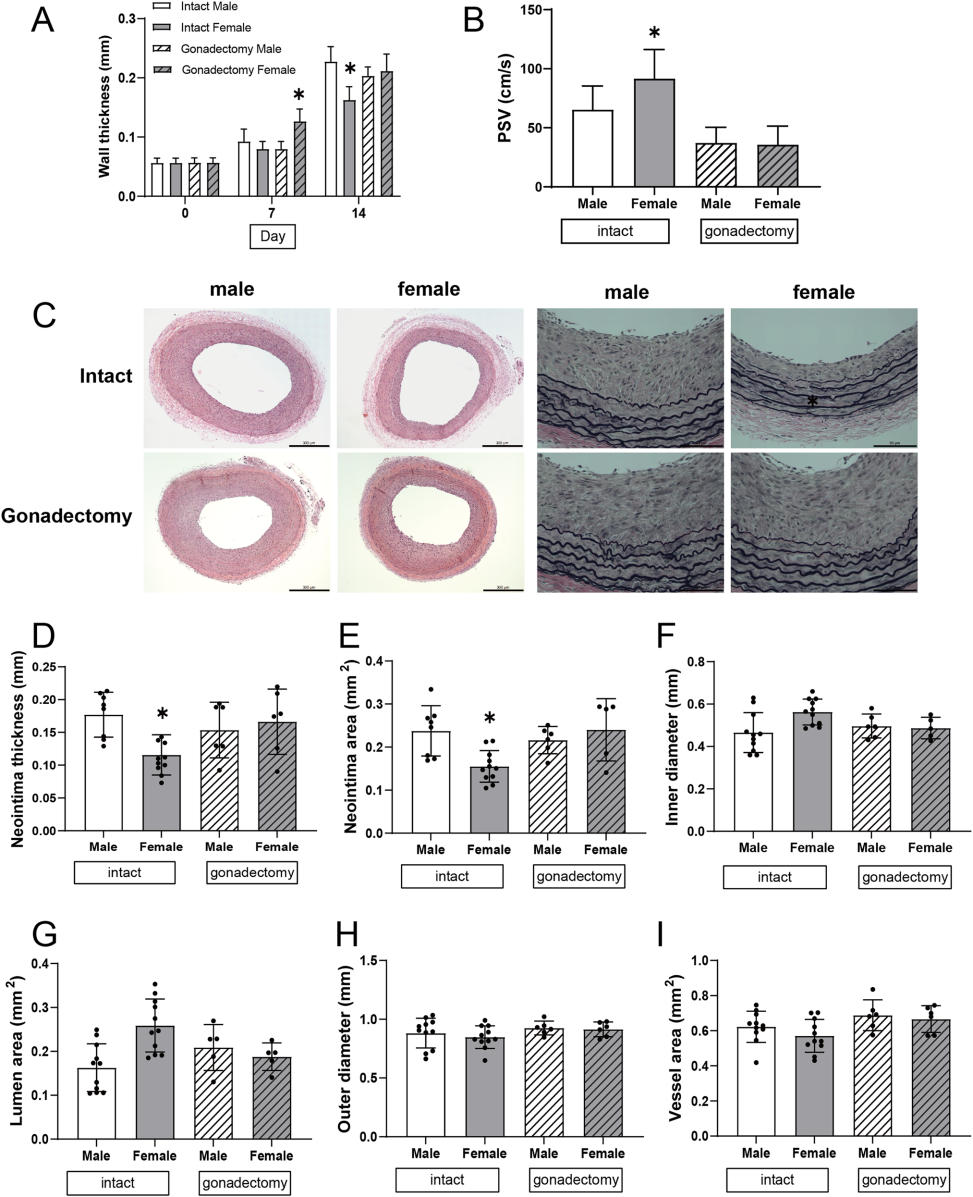
Sex differences after rat carotid balloon injury. **A** Bar graph shows wall thickness of carotid in rat after balloon injury with or without gonadectomy, n = 6–12, *p* < 0.0001 (ANOVA). Day 14, *p < 0.0001 (Sidak’s post hoc). **B** Bar graphs show sex differences of peak systolic velocity (PSV) of left common carotid in rat after balloon injury in intact and gonadectomy rats. intact, *p = 0.0269, gonadectomy, *p* = 0.9999; **C** Representative photomicrographs of left common carotid arteries after balloon injury in all groups at day 14. Hematoxylin and eosin (HE) stain (the left 4), scale bar, 300 μm; Elastin van Gieson (EVG) stain (the right 4), scale bar, 80 µm. **D**–**I** Bar graphs show sex differences of neointima thickness, neointima area, inner diameter, outer diameter, lumen area, vessel area of left common carotid in rat after balloon injury with or without gonadectomy, n = 6–12, unpaired t test. **D** neointima thickness: intact, *p = 0.0104, gonadectomy, *p* = 0.9932; **E** neointima area: intact, *p = 0.0069, gonadectomy, *p* = 0.8529; **F** inner diameter: intact, * *p* = 0.0231, gonadectomy, *p* = 0.9999; **G** lumen area: intact, * *p* = 0.0014, gonadectomy, *p* = 0.9247; **H** outer diameter: intact, *p* = 0.8437, gonadectomy, *p* = 0.9971; **I** vessel area: intact, *p* = 0.5265, gonadectomy, *p* = 0.9732

To evaluate the structural changes in the arterial wall after balloon injury, we measured the wall thickness and the diameter of balloon-injured carotid artery by ultrasound in vivo and correlated these findings with histology. Two weeks after balloon injury the arterial wall thickness was significantly increased in intact male rats, but less prominent among intact female rats (Figs. 1A, S2B); similarly, intact male rats showed decreased luminal diameter, with less decrease among female rats (Fig. S2C). Both female and male rats pretreated with gonadectomy had a uniformly increased wall thickness, associated with a corresponding decreased luminal diameter after balloon injury (Figs. 1A, S2C). The hemodynamic effect of these changes in the carotid artery was quantified by ultrasound. Two weeks after balloon injury of the left carotid artery, intact female rats had higher PSV and resistive indices compared to intact males and gonadectomized female and male rats (Figs. 1B, S2E, F); however, shear stress was similar between the groups (Fig. S2G). These data suggest that there are sex differences in the arterial response to injury significant enough to be detected by ultrasound.

Histology of the arteries 14 days after balloon injury showed significant thickening of the neointima, with multiple, circumferentially uniform smooth muscle cell (SMC) layers in the balloon-injured vessel, with less neointimal thickening in female rats (Fig. 1C, D); however, there were no significant differences in neointimal thickness between gonadectomized female and male rats (Fig. 1C, D). The increased neointimal thickness in intact male rats was associated with reduced inner diameter and lumen areas (Fig. 1F, G), with no sex differences in gonadectomized rats (Fig. 1C–G). In addition, there were no sex differences in the outer diameter or vessel areas across all groups, regardless of sex (Fig. 1H, I). These data are consistent with the ultrasound findings, that is intact female rats have less neointimal thickness after balloon injury, but this difference is not present after gonadectomy.

### Reduced SMC proliferation and matrix components after balloon injury in intact female rats

**Fig. 2:**
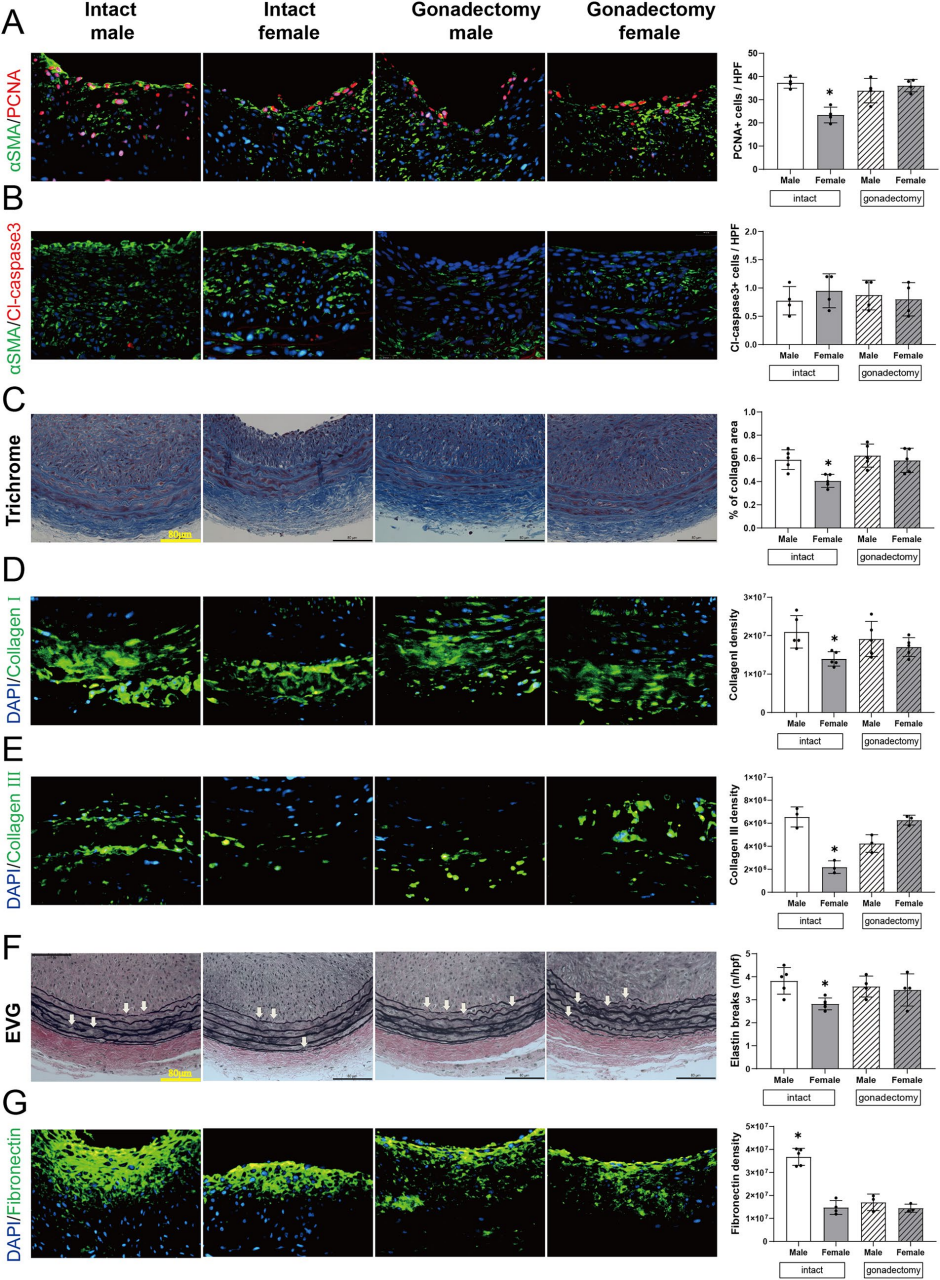
Sex differences in carotid artery wall composition after balloon injury. **A** Representative immunofluorescence of proliferating cell nuclear antigen (PCNA) in all groups. red, PCNA; green, α-actin; blue, DAPI. Scale bar, 26 μm. Magnification, ×40. Bar graph shows quantifications of PCNA + α-actin + cells. n = 4. *p = 0.0183, Kruskal–Wallis test, *p = 0.0451 (Dunn post hoc). **B** Representative immunofluorescence of cleaved caspase-3; in all groups. red, cleaved caspase-3; green, α-actin; blue, DAPI. Scale bar, 26 μm. Magnification, ×40. Bar graph shows quantifications of cleaved caspase-3 + α-actin + cells. n = 4. *p* = 0.6630, Kruskal–Wallis test. **C** Representative photomicrographs of masson trichrome stain in all groups. Scale bar, 80 µm. Bar graph shows percentage of collagen area in masson trichrome stained carotid wall in rat. n = 5. *p = 0.0105, Kruskal–Wallis test, *p = 0.0126 (Dunn post hoc). **D**, **E** Representative immunofluorescence of type I or III collagen. Green, collagen I or III; blue, DAPI. Scale bar, 26 μm. Magnification, ×40. **D** Bar graph shows quantification of collagen I immunofluorescence, n = 5, *p = 0.0367, Kruskal–Wallis test, *p = 0.0300 (Dunn post hoc). **E** Bar graph shows quantification of collagen III immunofluorescence: n = 3, *p = 0.0014, Kruskal–Wallis test, *p = 0.0395 (Dunn post hoc). **F** Representative photomicrographs of Elastin van Gieson (EVG) stain in all groups. Scale bar, 80 µm. Arrowheads on each photomicrograph show representative points of elastin breaks. Bar graph shows the number of elastin breaks in EVG stained carotid wall in rat. n = 4–5, *p = 0.0469, Kruskal–Wallis test, *p* = 0.0620 (Dunn post hoc). **G** Representative immunofluorescence of fibronectin. Green, fibronectin; blue, DAPI. Scale bar, 26 μm. Magnification, ×40. Bar graph shows quantification of fibronectin immunofluorescence, n = 3–5, *p = 0.0053, Kruskal–Wallis test, *p = 0.0460 (Dunn post hoc). (Color figure online)

Since intact female rats show less neointimal thickness after balloon injury, we examined the arterial walls for changes in both cells and extracellular matrix. Reduced wall thickness was characterized by decreased SMC proliferation in intact female rats but not in female rats pretreated with gonadectomy (Figs. 2A, S3A); apoptosis was uniformly low, with no significant differences between any groups (Figs. 2B, S3B). Decreased wall thickness was also associated with reduced deposition of several components of the extracellular matrix; percent of collagen area, both type I and type III collagen deposition were significantly lower in intact female rats (Fig. 2C–E). In addition, intact female rats had fewer elastin breaks (Fig. 2F) and less fibronectin immunoreactivity (Fig. 2G) compared to intact male rats. There were no significant sex differences in collagen deposition, elastin breaks or fibronectin immunoreactivity in female and male rats pretreated with gonadectomy. This data shows reduced cell proliferation and less accumulation of matrix components in female rats after balloon injury, but not in female rats pretreated with gonadectomy, consistent with the decreased neointimal thickening after balloon injury in female rats.

### Reduced M1-type and increased M2-type macrophages after balloon injury in intact female rats

**Fig. 3:**
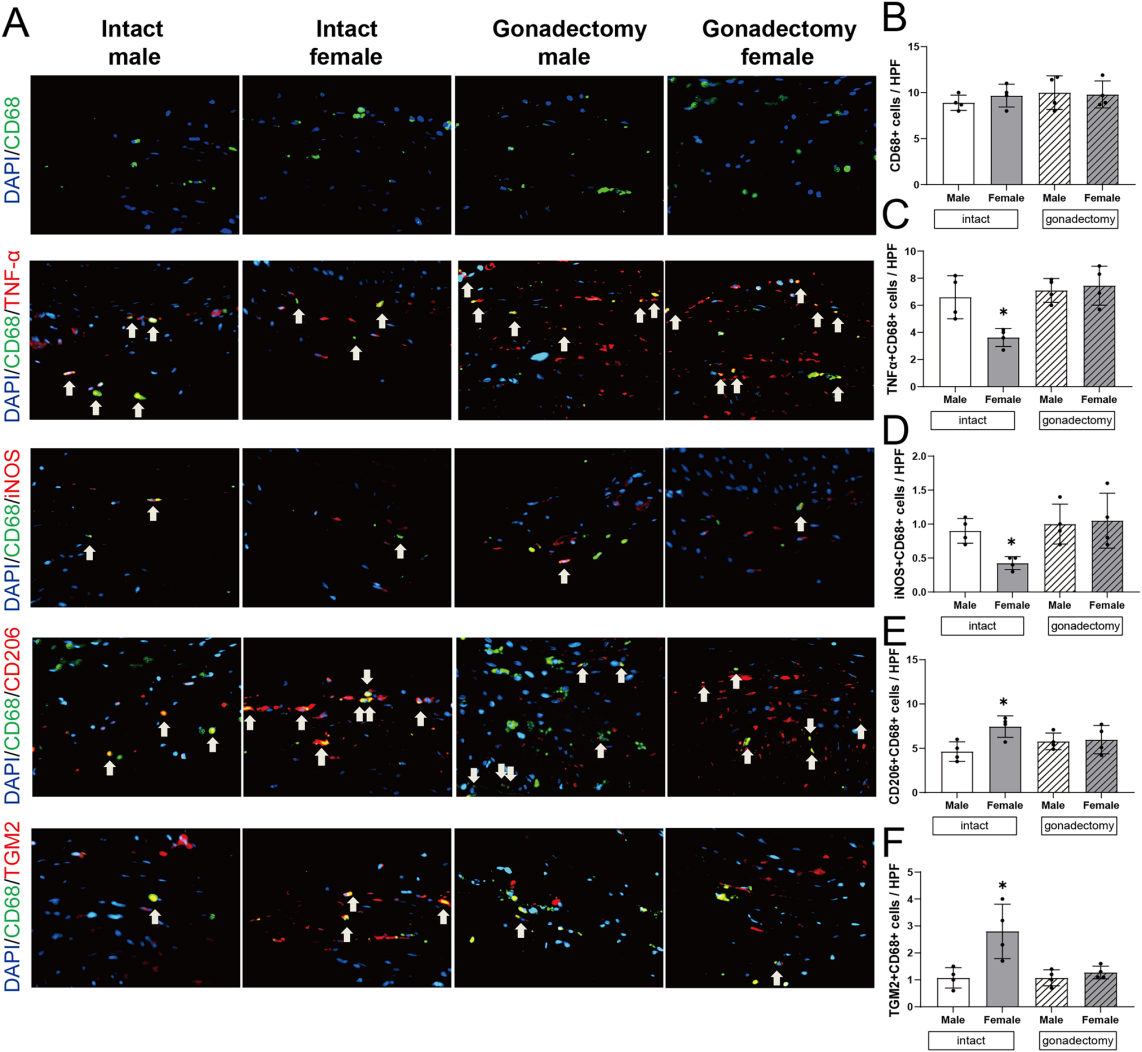
Sex differences in macrophage accumulation after balloon injury. **A** Representative photomicrographs showing immunofluorescence for CD68 (green), CD68 (green) and tumor necrosis factor-α (TNF-α; red), CD68 (green) and iNOS (red), CD68 (green) and CD206 (red), CD68 (green) and TGM2 (red) in the carotid wall in rats treated with balloon injury at days 14 with or without gonadectomy. Arrowheads on each photomicrograph show representative dual positive cells. Scale bar, 26 µm. **B** Bar graph shows quantifications of CD68+ cells in the carotid at day 14. n = 4. *p* = 0.4791, ANOVA. **C** Bar graph shows quantifications of CD68+ TNF-α+ cells in the carotid at day 14. n = 4. *p = 0.0199, ANOVA, *p = 0.0199 (Sidak’s post hoc). **D** Bar graph shows quantifications of CD68+ iNOS+ cells in the carotid at day 14. n = 4. *p = 0.0250, ANOVA, *p = 0.0297 (Sidak’s post hoc). **E** Bar graph shows quantifications of CD68+ CD206+ cells in the carotid at day 14. n = 4. *p = 0.0495, ANOVA, *p = 0.0321 (Sidak’s post hoc). **F** Bar graph shows quantifications of CD68+ TGM2+ cells in the carotid at day 14. n = 4. *p = 0.0027, ANOVA, *p = 0.0052 (Sidak’s post hoc). (Color figure online)

Since inflammation and macrophage-mediated responses are an important component of neointimal proliferation [19–21], we determined the sex differences in macrophage numbers and subtypes after arterial injury. There were no sex differences in accumulation of CD68+ cells among the intact or gonadectomy-treated rats (Fig. 3A, B). However, there was significantly decreased accumulation of CD68/TNFα dual-positive cells as well as CD68/iNOS dual-positive cells in intact female rats compared to intact male rats or among the gonadectomized rats (Figs. 3A, C, D, S4A, B), suggesting fewer M1-type macrophages in intact female rats. In addition, there were increased numbers of CD68/CD206 dual-positive cells as well as CD68/TGM2 dual-positive cells in the walls of intact female rats compared to intact male and gonadectomy-treated rats (Figs. 3A, E, F, S4C, D), consistent with relatively increased numbers of M2-type macrophages in intact female rats. These data are consistent with sex differences in the inflammatory and/or immune response in rats after balloon injury.

### Sex differences in arterial identity after balloon injury

**Fig. 4:**
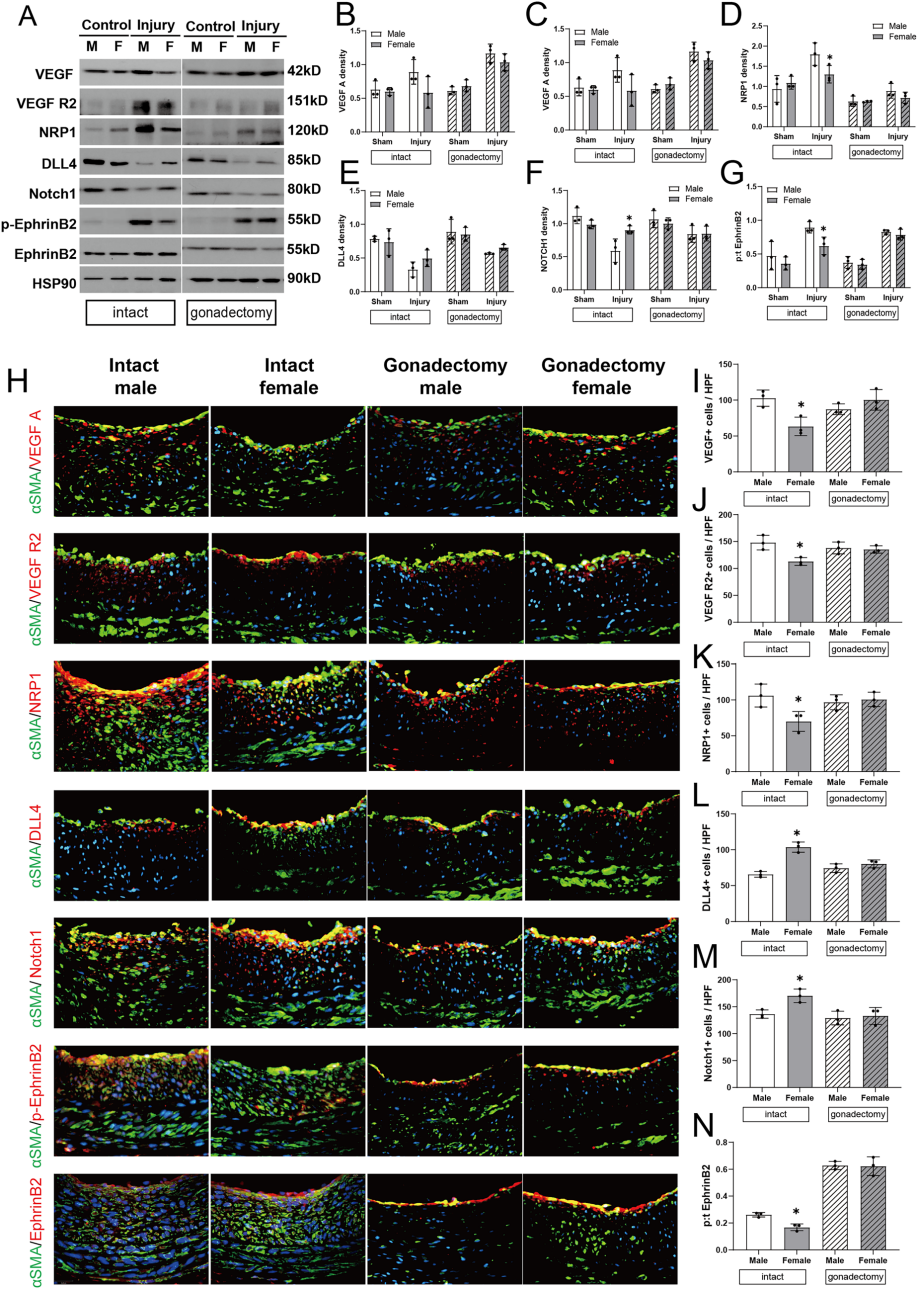
Sex differences in arterial identity after balloon injury. **A** Representative immunoblotting for arterial identity of VEGF-A, VEGF R2, NRP1, DLL4, Notch1 and phospho- or total (p:t) EphrinB2 in carotid artery of male and female rats treated with balloon injury or control, with or without gonadectomy, day 14, n = 3. **B**–**G** Bar graphs show quantification of densitometry (ratio of identity protein to HSP90). n = 3, two-way ANOVA, Sidak’s post hoc. **B** VEGF-A: intact, p = 0.0598, gonadectomy, p = 0.7043; **C** VEGF R2: intact, *p* = 0.1501, gonadectomy, *p* = 0.0833; **D** NRP1: intact, * *p* = 0.0332, gonadectomy, *p* = 0.7765; **E** DLL4: intact, *p* = 0.3654, gonadectomy, *p* = 0.8301; **F** Notch1: intact, *p = 0.0168, gonadectomy, *p* = 0.9999; **G** p:t EphrinB2: intact, *p = 0.0308, gonadectomy, *p* = 0.9853. **H** Representative merged immunofluorescence confirms the sex differences in VEGF-A, VEGF R2, NRP1, DLL4, Notch1 and p:t EphrinB2 expression in carotid artery after balloon injury in all groups. Intact male (first column), intact female (second column), gonadectomy male (third column), gonadectomy female (fourth column); from top to bottom, VEGF-A, VEGF R2, NRP1, DLL4, Notch1, p-EphrinB2 and EphrinB2 (red), α-actin (green), DAPI (blue); day 14. Scale bar, 26 μm. Magnification, ×40. **I**–**N** Bar graphs show quantification of immunofluorescence. n = 3, ANOVA, Sidak’s post hoc. **I** VEGF-A: *p = 0.0124 ANOVA, *p = 0.0148 (post hoc); **J** VEGF R2: *p = 0.0154 ANOVA, *p = 0.0116 (post hoc); **K** NRP1: *p = 0.0358 ANOVA, *p = 0.0358 (post hoc); **L** DLL4: *p = 0.0002 ANOVA, *p = 0.0002 (post hoc); **M** Notch1: *p = 0.0127 ANOVA, *p = 0.0414 (post hoc); **N** p:t EphrinB2: *p < 0.0001 ANOVA, *p < 0.0001 (post hoc). (Color figure online)

Since vessel identity can be altered in adults, including increased EphrinB2 expression in veins exposed to the fistula environment [22, 23], we determined whether there are differences in EphrinB2 expression after balloon injury and if there are sex differences in these changes. The baseline expression of EphrinB2 in the carotid artery was similar before balloon injury between female and male animals (Fig. 4A). Examination of balloon-injured arteries using Western blot showed increased VEGF-A, VEGFR2, Neuropilin-1 (NRP1) and phosphorylated EphrinB2 immunoreactivity compared to control vessels, with less increased VEGF-A, NRP-1 and phosphorylated EphrinB2 in intact female rats compared to intact male rats; these sex differences were not present in rats pretreated with gonadectomy (Fig. 4A–D, G). After balloon injury there was reduced Delta-like Ligand 4 (DLL4) and Notch1 immunoreactivity, with less decrease in intact female rats (Fig. 4A, E, F).

Immunofluorescence of the balloon-injured arteries confirmed the sex differences detected using Western blot, with reduced VEGF-A, VEGFR2, NRP-1 and phosphorylated EphrinB2 immunoreactivity in intact female rats compared to intact male rats, and these differences were not observed in rats pretreated with gonadectomy (Figs. 4H–N, S5). Of note, there were no sex differences in immunoreactivity of total EphrinB2 between any groups. These data are consistent with changes in the expression of markers of arterial identity after balloon injury, as well as sex differences in these changes in identity.

**Fig. 5:**
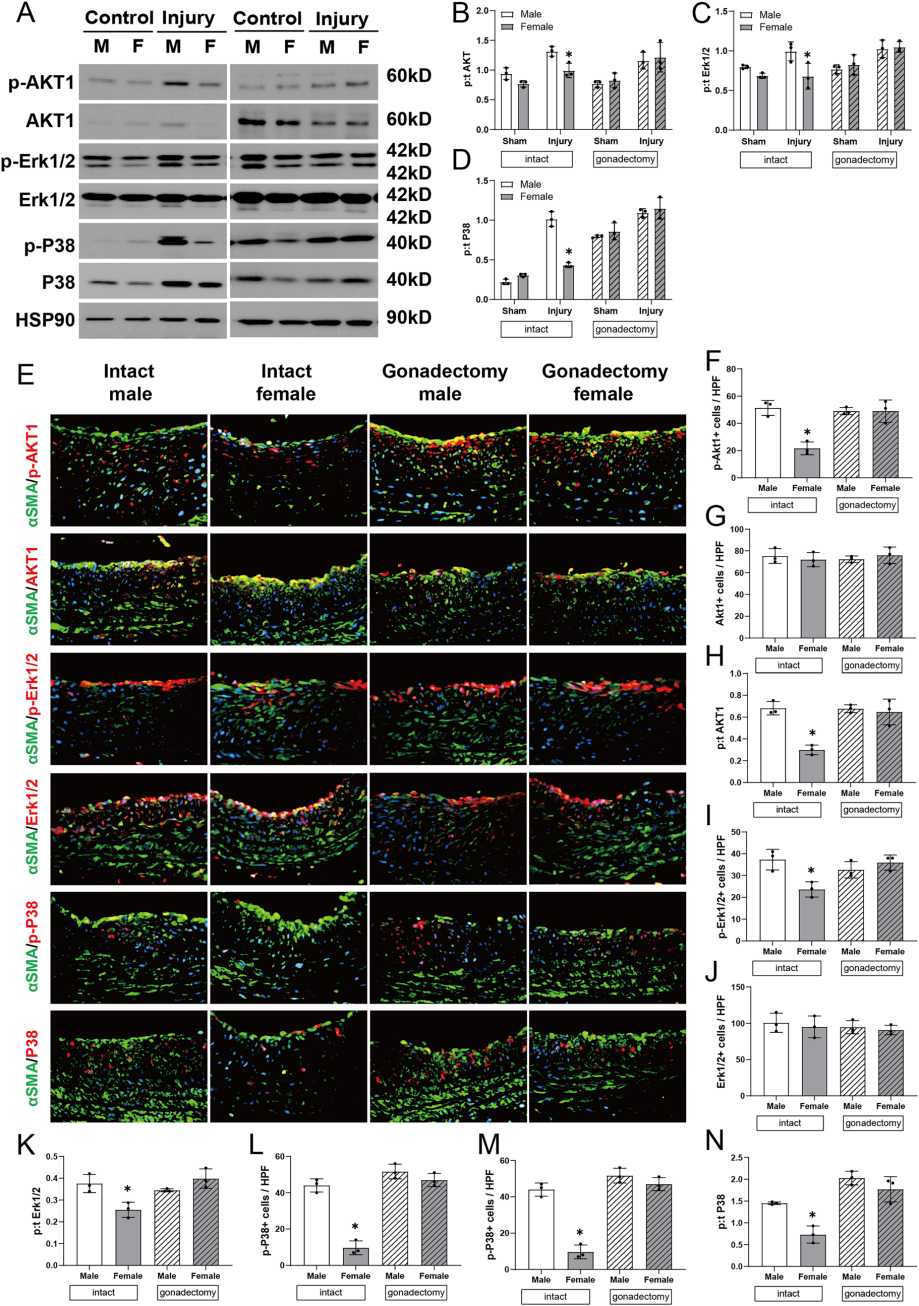
Sex differences in signaling pathways after balloon injury. **A** Representative immunoblotting of phospho- or total Akt1, ERK1/2 and p38 in carotid artery of male and female rats treated with balloon injury or control, with or without gonadectomy, day 14, n = 3. **B**–**D** Bar graphs show quantification of densitometry (ratio of phospho- to total protein). n = 3, two-way ANOVA, Sidak’s post hoc. **B** Akt1: intact, *p = 0.0296, gonadectomy, *p* = 0.9745; **C** ERK1/2: intact, *p = 0.0049, gonadectomy, *p* = 0.9975; **D** p38: intact, *p < 0.0001, gonadectomy, *p* = 0.8385. **E** Representative merged immunofluorescence confirms the sex differences in phospho- or total Akt1, ERK1/2 and p38 in carotid artery after balloon injury in all groups. Intact male (first column), intact female (second column), gonadectomy male (third column), gonadectomy female (fourth column); from top to bottom, p-Akt1, Akt1, p-ERK1/2, ERK1/2, and p-P38, P38 (red), α-actin (green), DAPI (blue); day 14. Scale bar, 26 μm. Magnification, ×40. **F**–**N** Bar graphs show quantification of immunofluorescence. n = 3, one-way ANOVA, Sidak’s post hoc. **F** p-Akt1: *p = 0.0006; **G** Akt1: *p* = 0.8096; **H** p:t Akt1: *p = 0.0005 ANOVA, *p = 0.0009 (post hoc); **I** p-ERK1/2: *p = 0.0105; **J** ERK1/2: *p* = 0.7663; **K** p:t ERK1/2: *p = 0.0052 ANOVA, *p = 0.0134 (post hoc); **L** p-P38: *p < 0.0001; **M** P38: *p = 0.0016; **N** p:t P38: *p = 0.0002 ANOVA, *p = 0.0079 (post hoc). (Color figure online)

Since EphrinB2 signaling is mediated through several downstream signaling pathways including Akt1, Extracellular signal-regulated kinases (ERK)1/2 and p38 [10, 24], we determined if there are sex differences in these pathways that could be consistent with the sex differences in arterial identity markers (Fig. 5). Balloon injury was associated with increased immunoreactivity of phosphorylated Akt1, phosphorylated ERK1/2 and phosphorylated p38 on Western blot, with reduced immunoreactivity in intact female rats compared to intact male rats but not in rats pretreated with gonadectomy (Fig. 5A–D). Immunofluorescence confirmed reduced immunoreactivity of phosphorylated Akt1, phosphorylated ERK1/2 and phosphorylated p38 in intact female rats but not in rats pretreated with gonadectomy (Figs. 5E–N, S6). These data suggest that the sex differences in vessel identity after balloon injury are accompanied by similar sex differences in downstream signaling pathways.

## Discussion

The main finding from this study is that the arterial response to balloon injury differs by sex, with intact female rats showing less neointimal thickening and luminal area preservation than in age-matched intact male rats (Fig. 1). Reduced wall thickness in female rats is associated with decreased SMC proliferation and differences in the extracellular matrix (Fig. 2). There was also a shift in macrophages in the artery, with fewer numbers of M1-type and greater numbers of M2-type macrophages (Fig. 3). Markers of vessel identity were differentially regulated between female and male rats, including reduced phosphorylation of EphrinB2 as well as downstream signaling pathways in female rats (Figs. 4, 5). These sex-specific differences were absent in rats pretreated with gonadectomy. In toto, these data suggest that vessel identity is differentially regulated during the response to balloon injury and may be a novel mechanism regulating this response.

Despite continued advances in endovascular technology that have increased treatment options for patients with vascular disease, restenosis after endovascular therapy remains a major concern [25–27]. Although sex-specific outcomes after endovascular interventions are well known, younger women are relatively protected from PAD; however, by the seventh decade, the incidence of PAD in women ultimately surpasses that of men, suggesting an interaction between sex and age [28–33]. The number of preclinical studies directly comparing restenosis between the sexes is extremely limited relative to the large number of studies exploring mechanisms of restenosis [6]. An early report of sex differences in response to arterial injury showed significantly decreased SMC proliferation in female rats [34]. Additional studies have also shown decreased neointimal proliferation and increased luminal area in female rats [15, 35, 36]. Similarly, there is decreased activation of adventitial cells during neointimal proliferation in female rats, suggesting decreased accumulation of several components of the extracellular matrix [37, 38].

Our data are consistent with the above-mentioned studies, with less neointimal thickening and preservation of luminal area in female rats that is accompanied by decreased SMC proliferation (Figs. 1, 2). In addition, there was decreased deposition of type I and III collagen as well as diminished fibronectin in intact female rats (Fig. 2), which is consistent with neointimal proliferation and extracellular matrix collagen suppressed by 17β-estradiol [39]. We have previously shown that reduced collagen density is associated with venous outward remodeling in an arteriovenous fistula model [40] and reduced elastin breaks are associated with arterial outward remodeling in a pseudoaneurysm model [41]. In this study, despite decreased deposition of type I and III collagen and decreased elastin breaks in female rats, there was no sex difference in outward remodeling (Figs. 1, S2). These findings suggest that SMC proliferation and extracellular matrix production are associated with sex differences in inward remodeling but not in outward remodeling in this model.

Our primary new finding shows that phosphorylation of the arterial determinant EphrinB2 is increased after balloon injury (Fig. 4). However, there is less EphrinB2 phosphorylation in intact female rats but not in rats pretreated with gonadectomy (Fig. 4), which correlate with the amounts of neointimal hyperplasia; sex differences in other markers of vascular identity, such as VEGF and Notch, are similar (Fig. 4). The transmembrane-ligand EphrinB2 is an embryonic determinant of arterial identity, whereas its corresponding receptor EphB4 is a determinant of venous fate [7, 8]. Although EphrinB2-EphB4 function regulates venous remodeling [22, 23, 42, 43], the function of this pathway in arterial injury, as well as potential sex differences, have not been well characterized. Cyclic stretch increases expression of EphrinB2 in arterial endothelial cells [44], suggesting a function for EphrinB2 in the response to stretch injury such as occurs with balloon injury. These data suggest that markers of vessel identity, such as EphrinB2, are regulated during arterial remodeling and show sex-specific differences, suggesting a potential novel mechanism.

Our data also show sex differences in some components of the signaling pathway downstream of EphrinB2, with phosphorylation of Akt1, Erk1/2, and p38 following the same trend as that of EphrinB2 phosphorylation. Markers of vessel identity such as EphrinB2 are part of a signaling pathway that regulates SMC migration and monocyte recruitment, regulates blood pressure, and promotes arteriogenesis [44–46]. Although little has been described about the sex-specific regulation of EphrinB2 after arterial injury, sex differences in potential downstream mediators are also observed in this study (Fig. 5). Akt1, ERK1/2 and p38 have important roles of vascular proliferation, migration, remodeling and arterial gene expression [47, 48]. We previously showed that inhibition of the Akt1-mammalian target of rapamycin complex 1 (mTORC1) during venous remodeling decreases SMC and macrophage proliferation, increasing venous remodeling [49]; similarly, reduced venous wall thickening in Akt1 knockout mice suggests that EphrinB2-mediated Akt1 signaling participates in venous wall thickening and remodeling [22]. Since there are sex differences in phosphorylation of Akt1, ERK1/2 and p38 in the response to arterial balloon injury (Fig. 5), it is possible that these mediators that are downstream of EphrinB2 may be a mechanism by which EphrinB2 regulates arterial remodeling.

Macrophages have important roles in collagen production during vascular remodeling [40, 41]. We have previously shown that M2-type macrophages are critical for venous adaptive remodeling [40]. However, there are limited data describing sex differences in macrophage differentiation, polarization, and proliferation in response to arterial injury. We report that among the macrophages in the media after balloon injury, there were fewer M1-type macrophages and greater numbers of M2-type macrophages in the walls of intact female rats compared to intact males and with gonadectomy-treated rats (Fig. 3). These data suggest that the sex differences leading to differential macrophage phenotypic switching during arterial remodeling may contribute to the sex difference in the amount of neointima hyperplasia that occurs in the rat carotid balloon injury model.

Clinical studies as well as gonadectomized young animal models have shown that estrogen has vasoprotective effects in the cardiovascular system. We used gonadectomy to determine whether sex hormones could be a mechanism of the observed sex differences. The observed sex difference in neointima hyperplasia was diminished in rats treated with gonadectomy, consistent with clinical data such as lack of any significant association between sex and restenosis after carotid stenting in postmenopausal women [50]. Interestingly, all of the observed sex differences in this study, including EphrinB2 phosphorylation, collagen production, SMC proliferation and macrophage differentiation, were abolished in rats treated with gonadectomy, suggesting that sex hormones are a fundamental mechanism in the vascular response to injury.

This study has several limitations. Although our rat model of neointimal hyperplasia after balloon injury recapitulates restenosis after endovascular therapy, the role of patient comorbid conditions such as hypertension, diabetes and hyperlipemia that could affect neointimal hyperplasia were not examined in this model. We also did not administer individual sex hormones to rats pretreated with gonadectomy to evaluate the resultant impact on neointimal hyperplasia; however, the protective role of estrogens against neointimal hyperplasia has been shown in prior studies and our data is consistent with them. Similarly, the downstream mediators of EphrinB2 were not directly manipulated.

## Conclusions

Female rats have decreased neointimal hyperplasia after balloon injury compared to male rats, but not after gonadectomy; similarly, there is sex-specific regulation of vessel identity. These data support an association between vessel identity and the sex-differences in the response to arterial injury, and suggest sex-specific therapy for arterial restenosis.

## Perspectives and significance

Mechanisms for the sex differences in clinical outcomes after endovascular treatment of atherosclerotic arterial disease have not been established. This paper shows that markers of vessel identity are differentially regulated between female and male rats after carotid balloon injury, and the differences in arterial identity are dependent on intact sex hormones. Alteration of arterial identity may be a novel mechanism explaining sex differences in neointimal proliferation after arterial injury and suggest sex-specific therapy for arterial restenosis.

## Supplementary Information

Below is the link to the electronic supplementary material.

Supplementary file1 (PDF 1498 kb)

## Data Availability

Please contact the first or last author for data or materials requests.
