# Supplementary material for: Sex differences in arterial identity correlate with neointimal hyperplasia after balloon injury
Source: Mol Biol Rep. 2022 Jun 17;49(9):8301–15. doi: 10.1007/s11033-022-07644-2 (PMC9463237; doi:10.1007/s11033-022-07644-2)
Supplement: Supplementary file 1 — Supplementary file1 (PDF 1498 kb) [file 11033_2022_7644_MOESM1_ESM.pdf]

Supplement

Sex Differences in Arterial Identity Correlate with Neointimal Hyperplasia after Balloon Injury

Mingjie Gao[1,2,3], Xixiang Gao[2,3,4], Ryosuke Taniguchi[2,3,5], Anand Brahmandam[2,3], Yutaka Matsubara[2,3,6], Jia Liu[2,3], Hao Liu[2,3,7], Weichang Zhang[2.3], Alan Dardik[2,3,8*]

[1] Department of Vascular Ultrasonography, Xuanwu Hospital, Capital Medical University, Beijing, China

[2] Vascular Biology and Therapeutics Program, Yale School of Medicine, New Haven, CT, USA

[3] Division of Vascular and Endovascular Surgery, Department of Surgery, Yale School of Medicine, New Haven, CT, USA

[4] Department of Vascular Surgery, Xuanwu Hospital, Capital Medical University, Beijing, China

[5] Division of Vascular Surgery, The University of Tokyo, Tokyo, Japan

[6] Department of Surgery and Sciences, Kyushu University, Fukuoka, Japan

[7] Division of Vascular and Interventional Radiology, Department of General Surgery, Nanfang Hospital, Southern Medical University, Guangzhou, China

[8] Department of Surgery, VA Connecticut Healthcare Systems, West Haven, CT, USA

Supplementary Table 1 (Table S1). List of Antibodies.

Target antigen	Vendor or Source	Catalog #	Working concentration
α-actin	Abcam	ab5694	1:200
α-actin	Invitrogen	14-9760-82	1:200
Akt1	Cell Signaling	2967	IF 1:100, WB 1:1000
CD206 (mannose R)	Abcam	ab64693	1:100
Cleaved Caspase-3	Cell Signaling	9661	1:100
Collagen-1	Abcam	ab34710	1:100
Collagen-3	Abcam	ab7778	1:100
DLL4	Abcam	ab183532	WB 1:1000
DLL4	Novusbio	NB600-892	IF 1:100
EphrinB2	R&D Systems	AF496	IF 1:100
EphrinB2 (HRP)	Abcam	ab201512	WB 1:8000
EphrinB2 (phospho-Y316)	Abcam	ab119323	WB 1:1000
Erk1/2	Cell Signaling	9102	IF 1:100, WB 1:1000
Erk1/2 (phosphor-Thr202/Tyr204)	Cell Signaling	9101	IF 1:100, WB 1:1000
Fibronectin	Abcam	ab2413	1:100
GAPDH	Cell Signaling	2118	WB 1:10000
Goat IgG control	R&D systems	AB-108-C	Same as primary Antibodies
HSP90	Santa Cruz Biotechnology	sc-13119	WB 1:10000
IL-10	Abcam	ab9969	1:100
iNOS	Abcam	ab15323	1:100
Mouse IgG, Alexa Fluor 488	Invitrogen	A-21202	IF 1:400
Mouse IgG, Alexa Fluor 568	Invitrogen	A-11061	IF 1:100
Mouse IgG, HRP-linked Antibody	Cell Signaling Technology	7076	WB 1:5000
Mouse IgG control	Santa-Cruz	sc-2025	Same as primary Antibodies
Notch1	Invitrogen	PA5-95827	IF 1:100, WB 1:1000
NRP1	Abcam	ab81321	IF 1:100, WB 1:1000
p-Akt1	Cell Signaling	9018S	IF 1:100, WB 1:1000
P38	Cell Signaling	8690	IF 1:100, WB 1:1000
p38 (phosphor-Thr180/Tyr182)	Cell Signaling	4511	IF 1:100, WB 1:1000
PCNA	Santa Cruz Biotechnology	sc-56	1:100
Rabbit IgG, Alexa Fluor 488	Invitrogen	A-21206	IF 1:100
Rabbit IgG, Alexa Fluor 568	Invitrogen	A 10042	IF 1:100

Rabbit IgG, HRP-linked Antibody	Cell Signaling Technology	7074	WB 1:5000
Rabbit IgG control	Santa-Cruz	sc-3888	Same as primary Antibodies
Rat IgG control	BD Bioscience	559478	Same as primary Antibodies
TGM2	Cell Signaling	cst3557	1:100
TNF-a	Abcam	ab6671	1:100
VEGF-A	Santa Cruz	sc-152	IF 1:100, WB 1:1000
VEGF-R2	Abcam	ab39638	IF 1:100, WB 1:1000
vWF	Abcam	ab6994	1:200

IF: Immunofluorescence; WB: Western Blot; PCNA: Proliferating Cell Nuclear Antigen

Table S2. Baseline characteristics of male and female rats.

Items	Intact rats			Gonadectomy rats		
	**Male**	**Female**	***P* value**	**Male**	**Female**	***P* value**
SBP (mmHg)	109.8 ± 5.9	99.5 ± 4.1	0.185	104.5 ± 5.5	100.3 ± 4.5	0.937
DBP (mmHg)	81.67 ± 3.80	73.83 ± 3.11	0.142	79.2 ± 1.5	73.3 ± 1.7	0.442
Cholesterol (mg/dl)	89.33 ± 4.86	81.00 ± 5.35	0.276	88.01 ± 2.51	87.67 ± 4.35	0.998
LDL (mg/dl)	14.83 ± 2.52	11.67 ± 2.35	0.380	14.67 ± 1.89	14.00 ± 1.31	0.995
Triglycerides (mg/dl)	127.70 ± 15.36	98.51 ± 9.40	0.136	117.70 ± 6.93	105.50 ± 7.02	0.835
Glucose (mg/dl)	176.2 ± 7.56	164.8 ± 7.69	0.318	171.5 ± 4.73	170.0 ± 7.94	0.988
Stroke volume (ml)	0.181 ± 0.01	0.185 ± 0.01	0.799	0.162 ± 0.01	0.138 ± 0.01	0.345
Cardiac output (ml)	70.73 ± 5.38	67.07 ± 2.34	0.547	61.53 ± 4.18	50.12 ± 2.81	0.192
% EF (%)	76.29 ± 1.06	76.17 ± 1.57	0.949	72.67 ± 0.61	73.93 ± 1.94	0.917
% FS (%)	46.19 ± 1.07	46.19 ± 1.64	0.999	42.67 ± 0.61	45.57 ± 1.05	0.314
HR (bpm)	388 ± 12	364 ± 10	0.163	380 ± 14	364 ± 6	0.719

SBP: systolic blood pressure, DBP: diastolic blood pressure, LDL: low density lipoprotein,

EF: ejection fraction, FS: fractional shortening, HR: heart rate, bpm: beats per minute

Table S3. Baseline hemodynamic parameters in male and female rats.

Items	Intact rats			Gonadectomy rats		
	**Male**	**Female**	***P* value**	**Male**	**Female**	***P* value**
Diameter (mm)	0.853 ± 0.018	0.863 ± 0.020	0.721	0.910 ± 0.092	0.895 ± 0.126	0.814
Lumen area (mm^2)	0.576 ± 0.025	0.589 ± 0.028	0.721	0.656 ± 0.130	0.638± 0.175	0.849
Wall thickness (mm)	0.055 ± 0.002	0.056 ± 0.002	0.683	0.057 ± 0.008	0.057 ± 0.008	0.999
PSV (cm/s)	81.23 ± 11.08	81.09 ± 11.19	0.997	77.50 ± 9.46	72.33 ± 1.63	0.142
EDV (cm/s)	13.54 ± 3.82	12.36 ± 4.27	0.811	11.50 ± 2.51	8.83 ± 0.75	0.600
Resistive index	0.883 ± 0.012	0.851 ± 0.008	0.212	0.885 ± 0.023	0.874 ± 0.013	0.065
Spectral broadening index	0.560 ± 0.053	0.581 ± 0.067	0.436	0.604 ± 0.097	0.589 ± 0.102	0.432
Flow (mm^3/s)	27.78 ± 1.13	28.49 ± 1.36	0.690	30.06 ± 1.94	26.95 ± 3.07	0.412
SS (dynes/cm^2)	269.7 ± 12.5	265.8 ± 11.2	0.815	242.6 ± 21.8	223.2 ± 11.6	0.450

PSV: peak systolic velocity, EDV: end diastolic velocity, SS: shear stress

Supplementary Figure Legends

Fig. S1 Experimental design. (A) Flow chart showing the experimental design of this study and the number of animals used in each analysis. U/S: ultrasound; IF: immunofluorescence; WB: western blot; (B) A diagram of neointimal hyperplasia. The yellow dotted line indicates intima. (C) Calculation formulae for data acquired from ultrasound measurements. PSV: peak systolic velocity; EDV: end diastolic velocity; TAMV: time-averaged maximum velocity; r: radius (in centimeters); η: Blood viscosity, was assumed to be constant at 0.035 poise.

Fig. S2 Sex differences after rat carotid balloon injury. (A) Line graphs showing body weight in each group up to day 14, $*p < 0.001$ (ANOVA), n = 6-12, $*p = 0.0039$ (Sidak's post hoc); cumulative patency rate, $p = 0.5538$ (Log-rank), n = 6-12 and survival rate, $p = 0.9999$ (Log-rank), n = 6-12. (B) Representative two-dimensional greyscale ultrasonography images of the rat carotid at post-operative day 14 of groups with and without balloon injury and/or gonadectomy in both male and female sex, n = 6-12. (C) Bar graph shows inner diameter of carotid in rat after balloon injury with or without gonadectomy, n = 6-12, $p = 0.0208$ (ANOVA). Day 14, * $p = 0.0019$ (Sidak's post hoc), n = 6-12. (D) Bar graph shows outer diameter of carotid in rat after balloon injury with or without gonadectomy, n = 6-12, $p =0.8520$ (ANOVA). (E) Representative Doppler wave forms of the rat carotid at post-operative day 14 of groups with and without balloon injury and/or gonadectomy in both male and female sex, n = 6-12. (F-G) Bar graphs show sex differences of resistive index, shear stress

(SS) of left common carotid in rat after balloon injury in intact and gonadectomy rats. (F) resistive index: intact, *p = 0.002, gonadectomy, p = 0.6677; (G) SS: sex differences in intact rats, p = 0.2217, sex differences in gonadectomy rats, p = 0.7672. Intact, n = 11-12; gonadectomy, n = 6, unpaired t-test.

Fig. S3 Individual channels of immunofluorescence shown in Fig 2. (A) Representative photomicrographs showing PCNA (red) and αSMA (green), also with mouse and rabbit IgG negative control; (B) Representative photomicrographs showing Cl-caspase3 (red) and αSMA (green), also with mouse and rabbit IgG negative control. Magnification, 40x.

Fig. S4 Individual channels of immunofluorescence shown in Fig 3. (A) Representative photomicrographs showing TNF-α (red) and αSMA (green), also with rabbit and mouse IgG negative control; (B) Representative photomicrographs showing iNOS (red) and αSMA (green); (C) Representative photomicrographs showing CD206 (red) and αSMA (green); (D) Representative photomicrographs showing TGM2 (red) and αSMA (green). Magnification, 40x.

Fig. S5 Individual channels of immunofluorescence shown in Fig 4. Representative photomicrographs showing (A) VEGF (red) and αSMA (green), (B) VEGF-R2 (red) and αSMA (green), (C) NRP1 (red) and αSMA (green), (D) DLL4 (red) and αSMA (green), (E) Notch1 (red) and αSMA (green), (F) p-EphrinB2 (red) and αSMA

(green), (G) EphrinB2 (red) and αSMA (green), also with rabbit and mouse IgG negative control. Magnification, 40x.

Fig. S6 Individual channels of immunofluorescence shown in Fig 5. Representative photomicrographs showing (A) p-AKT (red) and αSMA (green), (B) AKT (red) and αSMA (green), (C) p-Erk (red) and αSMA (green), (D) Erk (red) and αSMA (green), (E) p-P38 (red) and αSMA (green), (F) P38 (red) and αSMA (green). Magnification, 40x.

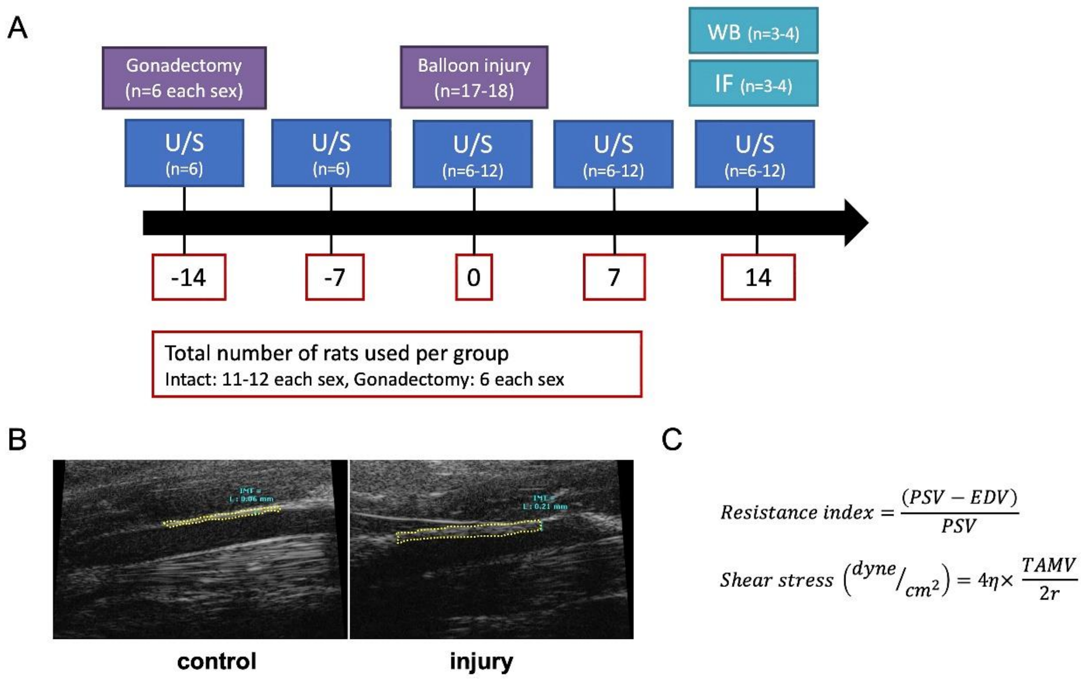

C

$$Resistance\ index = \frac{(PSV - EDV)}{PSV}$$

$$Shear\ stress\ \left({}^{dyne}/_{cm^2}\right) = 4\eta \times \frac{TAMV}{2r}$$

Fig. S1 Experimental design.

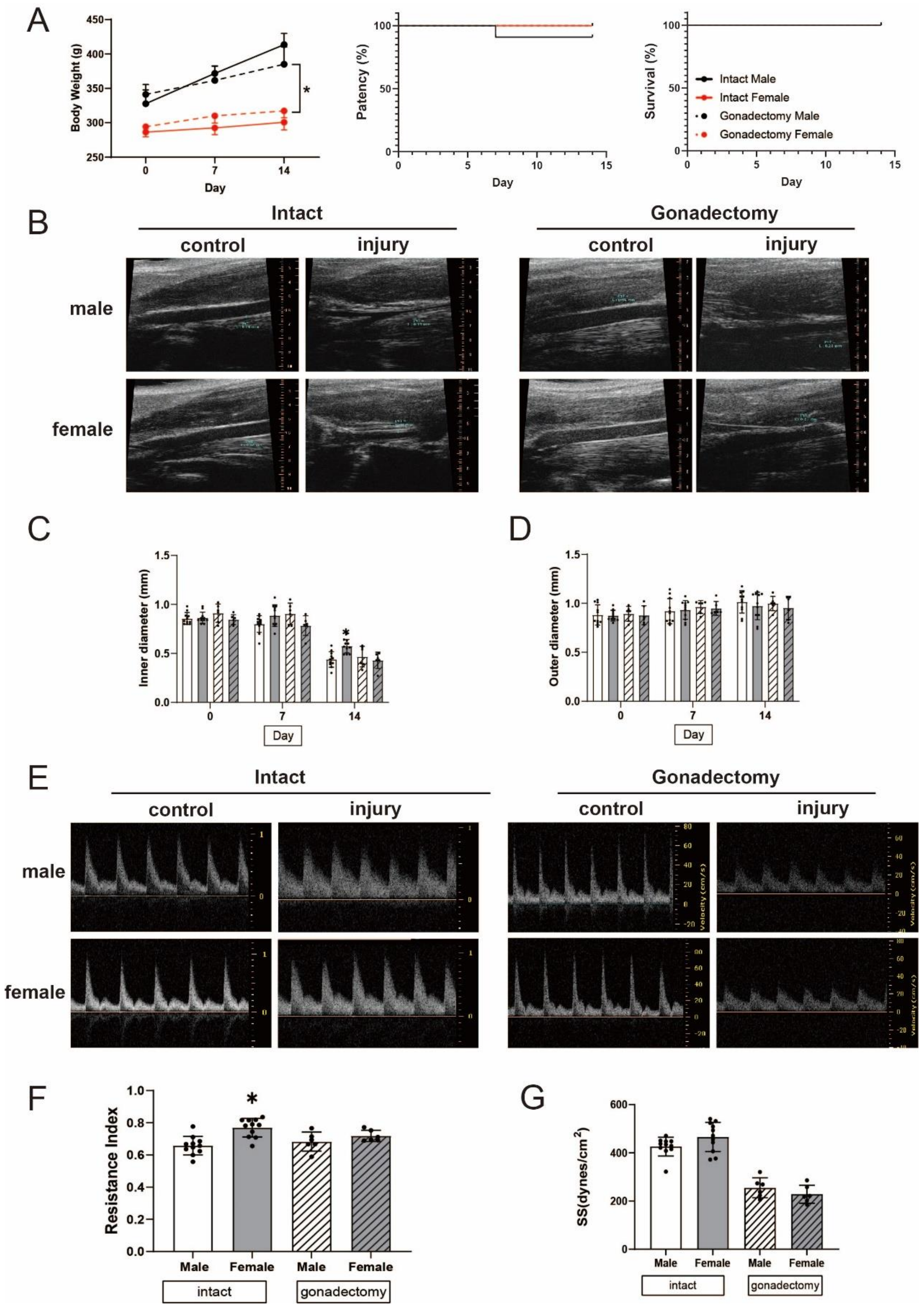

Fig. S2 Sex differences after rat carotid balloon injury.

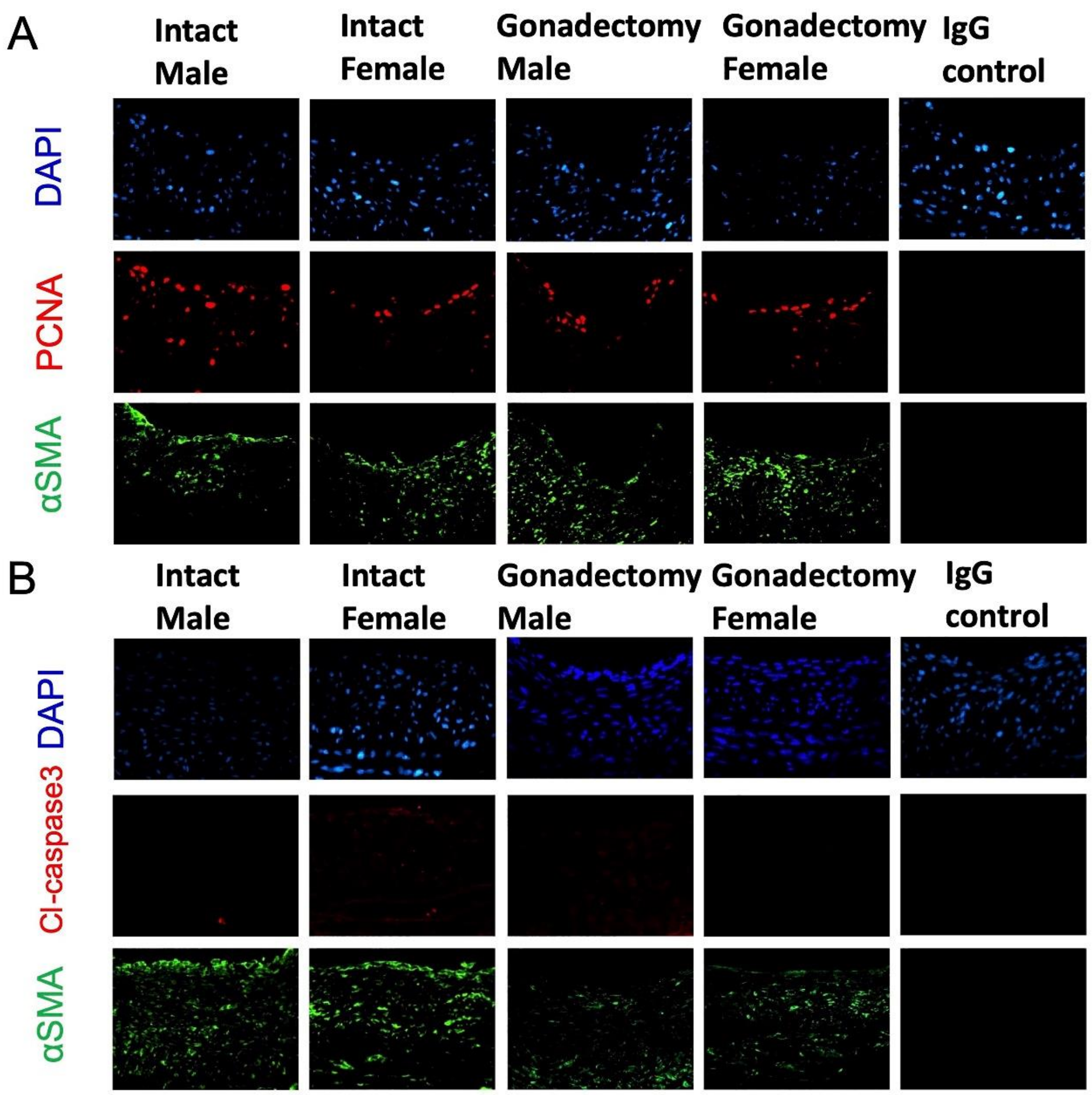

Fig. S3 Individual channels of immunofluorescence shown in Fig 2.

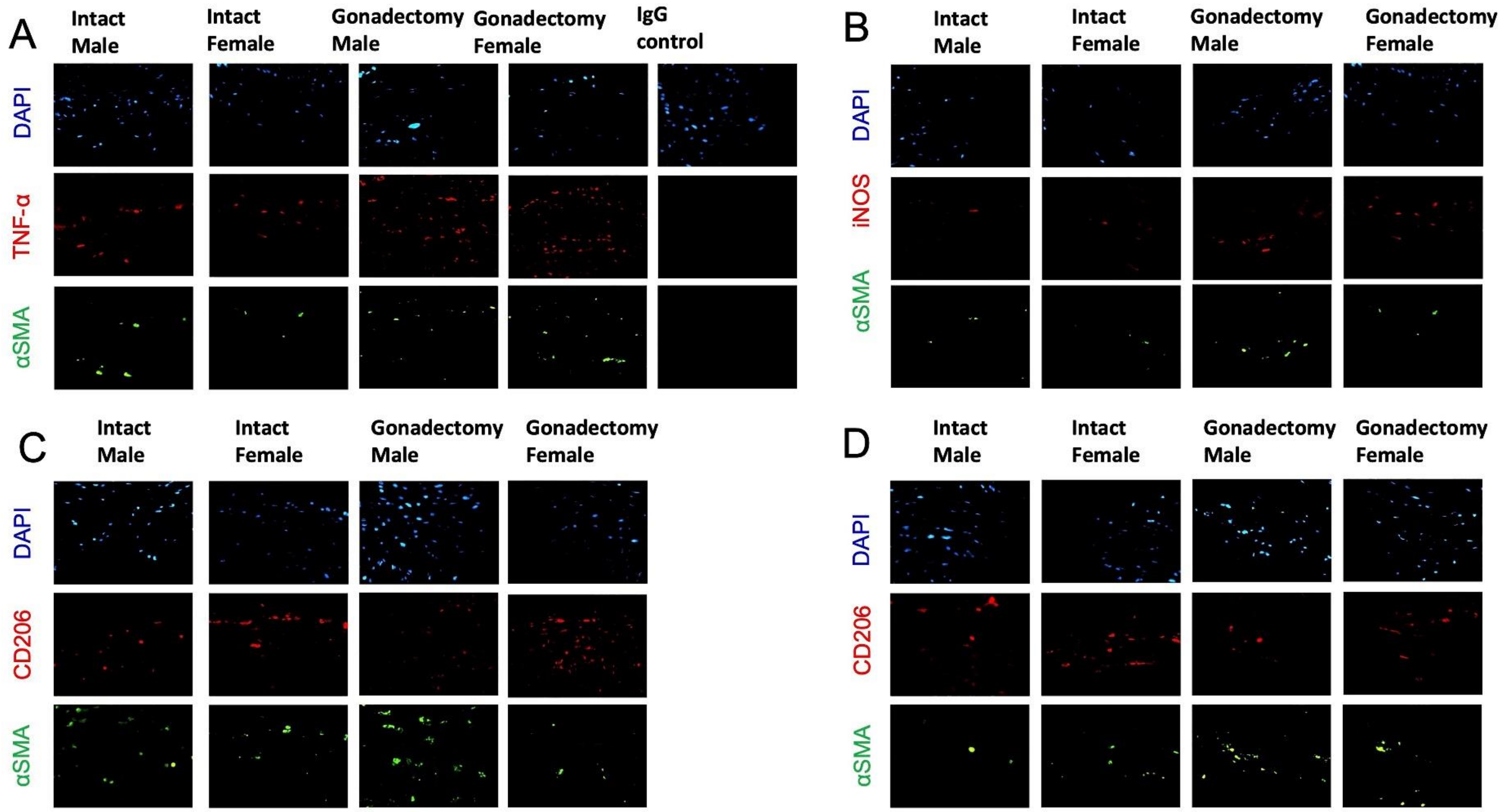

Fig. S4 Individual channels of immunofluorescence shown in Fig 3.

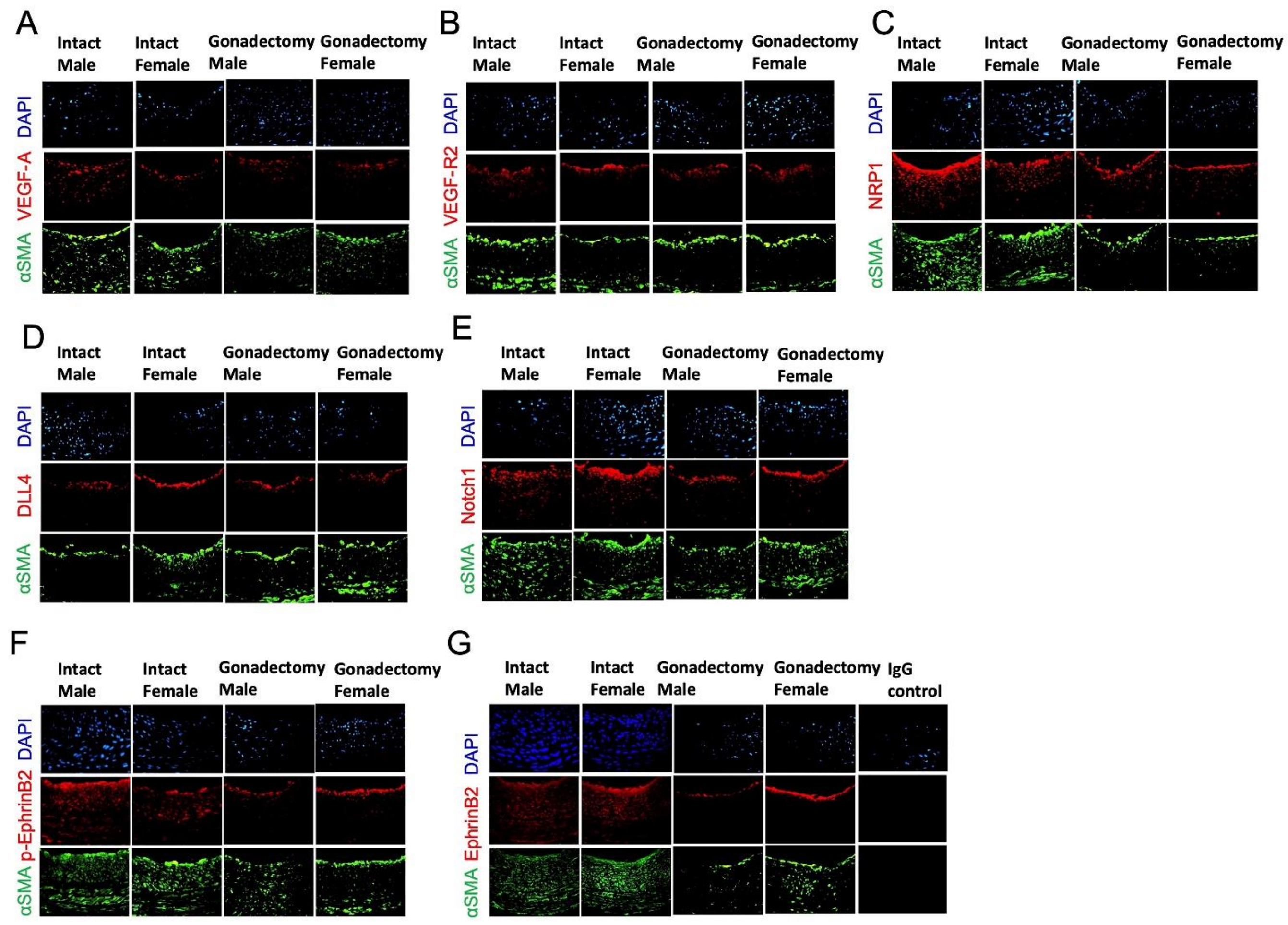

Fig. S5 Individual channels of immunofluorescence shown in Fig 4.

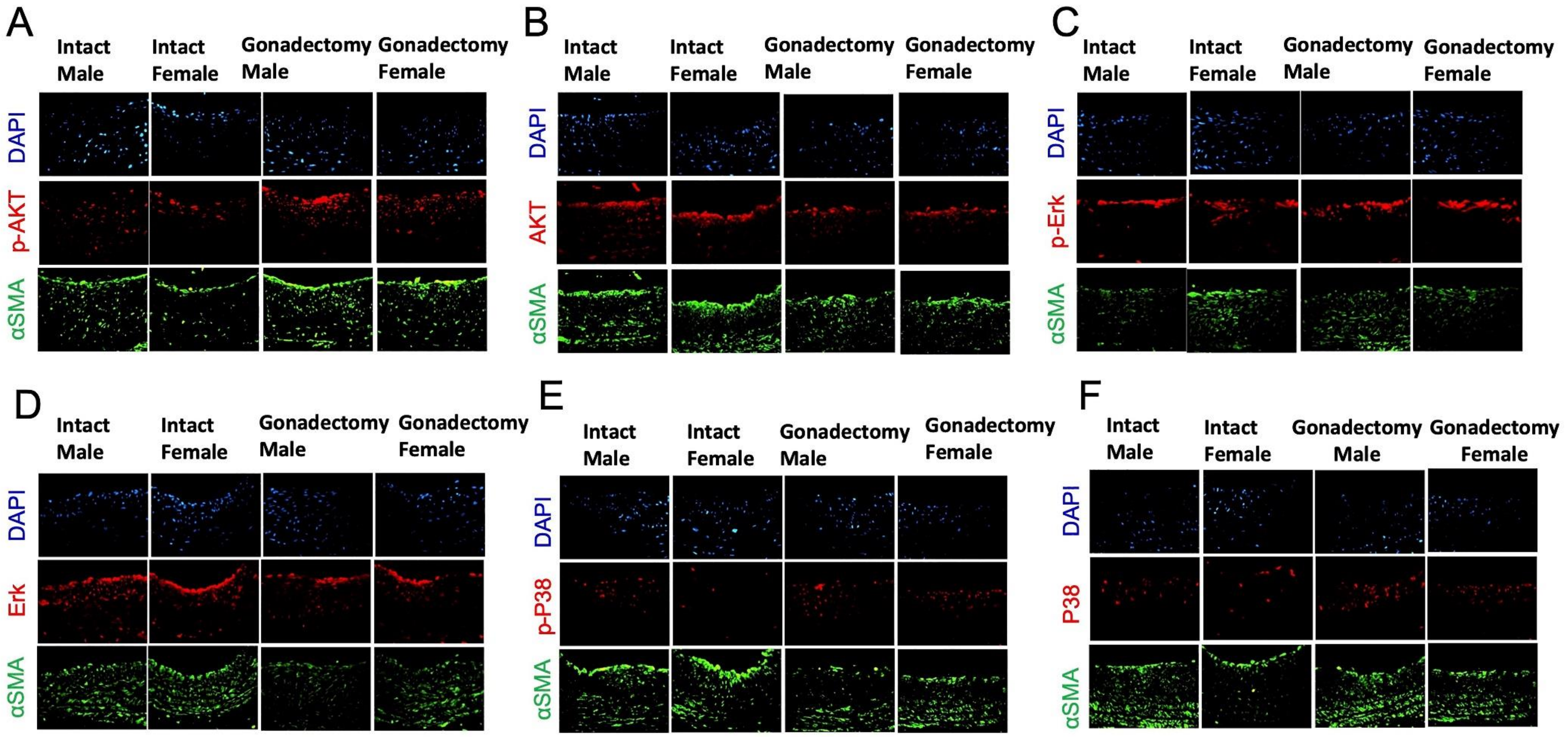

Fig. S6 Individual channels of immunofluorescence shown in Fig 5.
